# Improving the Photostability of Small-Molecule-Based Organic Photovoltaics by Providing a Charge Percolation Pathway of Crystalline Conjugated Polymer

**DOI:** 10.3390/polym12112598

**Published:** 2020-11-05

**Authors:** Jihee Kim, Chang Woo Koh, Mohammad Afsar Uddin, Ka Yeon Ryu, Song-Rim Jang, Han Young Woo, Bogyu Lim, Kyungkon Kim

**Affiliations:** 1 Department of Chemistry and Nano Science, Ewha Womans University, Seoul 03760, Korea; jhkim3664@ewhain.net (J.K.); ryuky@ewhain.net (K.Y.R.); 2 Department of Chemistry, Korea University, Seoul 136713, Korea; woocchang@korea.ac.kr (C.W.K.); soaibchebd@yahoo.co.uk (M.A.U.); 3 Future Technology Research Center, LG Sciencepark, LG Chem, 30, Magokjungang 10-ro, Gangseo-gu, Seoul 07796, Korea; songrimjang@lgchem.com; 4 Green Fine Chemical Research Center, Advanced Convergent Chemistry Division, Korea Research Institute of Chemical Technology (KRICT), 45 Jongga-ro, Jung-gu, Ulsan 44412, Korea

**Keywords:** organic solar cell, small molecule organic solar cell, stability

## Abstract

Photostability of small-molecule (SM)-based organic photovoltaics (SM-OPVs) is greatly improved by utilizing a ternary photo-active layer incorporating a small amount of a conjugated polymer (CP). Semi-crystalline poly[(2,5-bis(2-hexyldecyloxy)phenylene)-*alt*-(5,6-difluoro-4,7-di(thiophen-2-yl)benzo[*c*][1,2,5]thiadiazole)] (PPDT2FBT) and amorphous poly[(2,5-bis(2-decyltetradecyloxy)phenylene)-*alt*-(5,6-dicyano-4,7-di(thiophen-2-yl)benzo[*c*][1,2,5]thiadiazole)] (PPDT2CNBT) with similar chemical structures were used for preparing SM:fullerene:CP ternary photo-active layers. The power conversion efficiency (PCE) of the ternary device with PPDT2FBT (Ternary-F) was higher than those of the ternary device with PPDT2CNBT (Ternary-CN) and a binary SM-OPV device (Binary) by 15% and 17%, respectively. The photostability of the SM-OPV was considerably improved by the addition of the crystalline CP, PPDT2FBT. Ternary-F retained 76% of its initial PCE after 1500 h of light soaking, whereas Ternary-CN and Binary retained only 38% and 17% of their initial PCEs, respectively. The electrical and morphological analyses of the SM-OPV devices revealed that the addition of the semi-crystalline CP led to the formation of percolation pathways for charge transport without disturbing the optimized bulk heterojunction morphology. The CP also suppressed trap-assisted recombination and enhanced the hole mobility in Ternary-F. The percolation pathways enabled the hole mobility of Ternary-F to remain constant during the light-soaking test. The photostability of Ternary-CN did not improve because the addition of the amorphous CP inhibited the formation of ordered SM domains.

## 1. Introduction

Organic photovoltaics (OPVs) are currently receiving considerable attention because of their unique advantages, such as being lightweight and flexible [1,2,3]. Small-molecule (SM) organic semiconductor donor materials possess many unique characteristics compared to conjugated polymer (CP) donor materials. They have versatile molecular structures, a well-defined molecular weight, a fine-tunable molecular energy level, and less batch-to-batch variations. Recently, a breakthrough has been achieved in the field of SM-OPVs: power conversion efficiencies (PCEs) of over 14% have been successfully demonstrated [4,5,6,7,8,9,10,11,12,13,14,15,16,17].

However, SM-OPVs have poor device stability under various operation conditions, including light soaking and damp heat. This issue should be addressed before their mass production and industrial applications. Although several studies on the stability of SM-OPVs have been recently conducted, more careful research is necessary to resolve this issue [18,19,20,21]. A key problem is the significant burn-in loss (rapid drop in solar cell parameters at the beginning of device operation), particularly the rapid drop in the open-circuit voltage (*V*_OC_) [22]. One of the possible causes is trap formation in the photoactive layer during light soaking [20,23,24]. Compared to polymers, smaller molecules are reorganized more readily and their local morphology is altered in the presence of external stress, such as heat or light. This may lead to divergence from the optimized bulk heterojunction (BHJ) and result in fill factor (*FF*) and *V*_OC_ losses. One example of a bulk morphology change is the phase separation of the SM and fullerenes in SM-BHJ blends, which would result in the formation of large pure donor and acceptor phase crystallites [25,26].

The charges generated by light absorption and exciton dissociation at the electron donor/electron acceptor interface must be transported via intramolecular and intermolecular transport mechanisms. Intramolecular transport includes charge delocalization within aromatic units and movement along the conjugated polymeric backbone. Intermolecular transport occurs through so-called “hopping” processes [27]. Because intermolecular transport via hopping takes longer than intramolecular transport, hopping is the rate-determining step for charge transporting, and increased hopping enhances the probability of charge trapping. The efficiency of the hopping process would be more critical for SM-OPVs than for CP-based OPVs. Therefore, the charge transport in SM-OPVs must be critically dependent on the degree of molecular orientation and morphological change. This implies that a subtle change in the molecular morphology by external stress could significantly alter the charge-carrier mobility in SM-OPVs, resulting in their poor device stability. 

The addition of a CP to the active layer of SM-OPVs could lead to the formation of an efficient pathway for charge transport and render SM-OPVs less sensitive to subtle morphological changes. There have been several studies on enhancing the performance of SM-OPVs through the addition of small amounts of polymers to binary blends of SM and fullerene. For example, Huang et al. introduced polystyrene (PS) into an SM: fullerene active layer and Renolds et al. added polydimethylsiloxane to control the morphology of a SM-OPV device and improve its PCE [28,29]. The addition of PS increased the solution viscosity and promoted the formation of interconnected SM domains. The addition of CPs is preferable to the addition of non-conjugated polymers for enhancing the charge-carrier mobility and morphological stability.

In this study, we investigated the effect of the crystallinity of CPs on the stability of SM-OPV devices. Semi-crystalline poly[(2,5-bis(2-hexyldecyloxy)phenylene)-*alt*-(5,6-difluoro-4,7-di(thiophen-2-yl)benzo[*c*][1,2,5]thiadiazole)] (PPDT2FBT) and amorphous poly[(2,5-bis(2-decyltetradecyloxy)phenylene)-*alt*-(5,6-dicyano-4,7-di(thiophen-2-yl)benzo[c][1,2,5]thiadiazole)] (PPDT2CNBT) were investigated as additives [30,31]. PPDT2FBT exhibits a superior chain planarity and strong intermolecular ordering via intramolecular and intermolecular noncovalent coulombic interactions [30]. By contrast, PPDT2CNBT [31] exhibits amorphous properties because of its tilted polymeric backbone due to large cyano substituents. A binary blend of an SM donor named LGC-D073 and the acceptor PC_71_BM was used as the photoactive layer. PPDT2FBT or PPDT2CNBT was added to the binary blend to prepare a ternary photoactive layer. The ternary SM-OPV device with PPDT2FBT (Ternary-F) exhibited a higher stability under one sunlight soaking compared to the ternary device with PPDT2CNBT (Ternary-CN) or binary device (Binary). It was observed that the addition of semi-crystalline PPDT2FBT increased the hole mobility and rendered the SM-OPV devices less sensitive to subtle morphological changes.

## 2. Materials and Methods

### 2.1. Materials

#### 2.1.1. Synthesis of LGC-D073

LGC-D073 was prepared according to our previous report [31].

#### 2.1.2. Synthesis of PPDT2FBT and PPDT2CNBT

PPDT2FBT and PPDT2CNBT were synthesized using the same procedures outlined in our previous reports [31]. The average molecular weight and polydispersity index of PPDT2FBT were 40 kDa and 2.1, respectively, and those of PPDT2CNBT were 26 kDa and 2.3, respectively.

#### 2.1.3. Other Materials

Poly(3,4-ethylenedioxythiophene):poly(styrene sulfonate) (PEDOT:PSS) solution (Heraeus Clevios P VP AI 4083) and PC_71_BM (EM Index, Seoul, Korea) were purchased and used as received. The solvents chlorobenzene (Tokyo Chemical Industry, Tokyo, Japan) and 1,8-diiodooctane (Tokyo Chemical Industry) were used as received.

### 2.2. Preparation of Solutions for Photoactive Layer

For the preparation of Binary, a binary solution was prepared by dissolving 10 mg of LGC-D073 and 15 mg of PC_71_BM in 1 mL of a co-solvent of chlorobenzene and 1,8-diiodooctane in a weight ratio of 88:12. The solution was stirred at 80 ℃ for over 5 h before spin casting. For preparing Ternary-F and Ternary-CN, a binary solution containing PPDT2FBT (or PPDT2CNBT) and PC_71_BM in a weight ratio of 1:1 was first prepared with a PPDT2FBT (or PPDT2CNBT) concentration of 33 mg∙mL^−1^ in the cosolvent used for preparing Binary. The binary solution was stirred at 80 ℃ for 5 h and then blended with the LGC-D073:PC_71_BM binary solution used to prepare the Binary. The blending ratio of the LGC-D073:PC_71_BM binary solution to the PPDT2FBT (or PPDT2CNBT):PC_71_BM binary solution was 95:5 by volume. The ternary solutions were stirred at 80 ℃ for 1 h before casting the films. 

The prepared solutions were used for fabricating the photoactive layer of SM-OPV devices with the inverted structure of indium tin oxide (ITO)/ZnO/photoactive layer/MoO_3_/Ag. First, patterned ITO-coated glass substrates (20 Ω/sq) were ultrasonicated in a detergent, isopropyl alcohol (IPA), acetone, and IPA for 10 min and dried in a convection oven at 80 ℃ for 10 min. Subsequently, the cleaned substrates were treated in ultraviolet (UV) ozone for 20 min. A ZnO sol-gel precursor solution was spin-coated onto the substrate at a speed of 4000 rpm and hydrolyzed on a hot plate at 200 ℃ for 1 h. The binary or ternary solutions prepared for fabricating the photoactive layer were spin-coated onto a ZnO-coated ITO glass substrate. The spinning speed was varied from700 to 1500 rpm to obtain the optimum thickness around 80 nm. When the film thickness was thicker than the optimum thickness, the fill factor of the device was decreased. The films were annealed at 80 ℃ on a hot plate for 30 min. When the annealing temperature was higher than the optimized temperature, the domain size was increased and the device performance was decreased. Furthermore, 10 nm-thick molybdenum oxide (MoO_3_) and 100 nm-thick Ag electrode were evaporated in vacuum at a pressure of 3.0 × 10^−6^ Torr. The active area of the devices was 0.20 cm^2^. Finally, the devices were encapsulated using a UV resin, XNR 5570-B1 (Nagase ChemteX, Osaka, Japan).

### 2.3. Characterization

The current density and voltage of the OPVs were measured using a Keithley 2400 SourceMeter under AM 1.5 G irradiation (100 mW∙cm^−2^); a 150 W xenon-lamp-based solar simulator (McScience, Seoul, South Korea) was used for irradiating the OPV devices. The external quantum efficiency (EQE) of the OPVs was measured using a K3100 EQX IPCE measurement system (McScience, Seoul, South Korea) with a 300 W xenon lamp. UV-visible absorption spectra were obtained using a UV-2450 (Shimadzu, Shimane, Japan) spectrophotometer. Grazing incidence wide-angle X-ray scattering (GIWAXS) measurements were performed at the PLS-II 9A U-SAXS beamline of the Pohang Accelerator Laboratory (Pohang, South Korea). The photostability of the encapsulated devices was determined by light soaking them under 1 sunlight intensity. A 150 W metal-halide lamp was used for the light-soaking test. A BAS 100B electrochemical analyser was employed to obtain cyclic voltammetry (CV) data.

## 3. Results and Discussion

**Figure 1:**
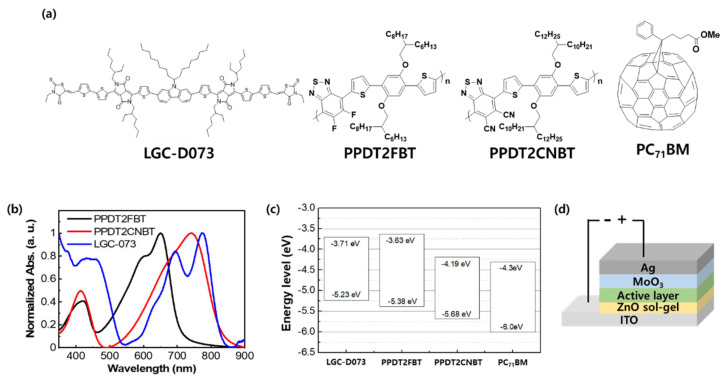
(**a**) Chemical structures, (**b**) thin-film absorption spectra, (**c**) energy levels diagram of photoactive materials, and (**d**) device structure of small molecule organic photovoltaic (SM-OPV).

Figure 1a shows the chemical structures of the materials used for preparing the photoactive layer of Binary, Ternary-F, and Ternary-CN. The thin-film absorption spectra of photoactive materials are shown in Figure 1b. A photoactive layer composed of LGC-073 and PC_71_BM was used for the fabrication of Binary. PPDT2FBT and PPDT2CNBT were added to the binary solution and used for fabricating Ternary-F and Ternary-CN, respectively. For the preparation of Binary, a binary solution was prepared by dissolving 10 mg of LGC-D073 and 15 mg of PC_71_BM in 1 mL of a cosolvent of chlorobenzene and 1,8-diiodooctane in a weight ratio of 88:12. For preparing Ternary-F and Ternary-CN, a binary solution containing PPDT2FBT (or PPDT2CNBT) and PC_71_BM in a weight ratio of 1:1 was first prepared with a PPDT2FBT (or PPDT2CNBT) concentration of 33 mg∙mL^−1^ in the cosolvent used for preparing the Binary. Then, the binary solution was blended with the LGC-D073:PC_71_BM binary solution used to prepare the Binary. The blending ratio of the LGC-D073:PC_71_BM binary solution to the PPDT2FBT (or PPDT2CNBT):PC_71_BM binary solution was 95:5 by volume. While these CPs have the same backbone, the F substituents on the benzothiadiazole units of PPDT2FBT were replaced by CN groups for preparing PPDT2CNBT. This caused a downward energy level shift for both the highest occupied molecular orbital (HOMO) and the lowest unoccupied molecular orbital, from −5.38 to −5.68 eV and from −3.63 to −4.19 eV for PPDT2FBT and PPDT2CNBT, respectively. Consequently, the optical bandgap of PPDT2CNBT (1.49 eV) was smaller than that of PPDT2FBT (1.75 eV) (Figure 1c). [31] With these materials, we fabricated SM-OPV devices with the inverted structure shown in Figure 1d.

**Figure 2:**
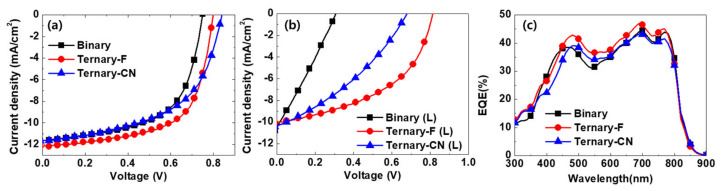
Current density vs. voltage curves for the fabricated SM-OPV devices (**a**) before and (**b**) after 1 sunlight soaking for 1500 h. (**c**) External quantum efficiency (EQE) spectra before the light soaking.

The current density–voltage (*J–V*) characteristics of the inverted-type solar cells are shown in Figure 2a and Table 1. The EQE spectra (Figure 2c) match well with the *J*_SC_ values. For Binary, the best PCE obtained was 5.17% for a *V*_OC_ of 0.75 V, a *J*_SC_ of 11.64 mA∙cm^−2^, and a *FF* of 0.59. The addition of PPDT2FBT to the binary blend enhanced the PCE of Ternary-F by up to 6.09%. Ternary-CN exhibited a PCE of 5.29%, which is higher than that of Binary because of the increased *V*oc. The increased *V*oc is ascribed to the deeper HOMO level of PPDT2CNBT as shown in Figure 1b. Despite its higher *V*oc, the PCE of Ternary-CN was not higher than that of Ternary-F. This is because of the low *FF*. Our previous report on the polymer OPV device with PPDT2CNBT and PC_71_BM revealed that the low *FF* is mainly due to the inefficient charge transport in PPDT2CNBT, which results in a high recombination rate and poor solar cell performance [31]. It may be ascribed to that the optimal charge transport pathway formed by LGC-D073 and PC_71_BM was interrupted and charge recombination sites increased following the addition of PPDT2CNBT.

First, the thermal stability of SM-OPV devices was investigated by applying an 80 °C thermal stress for 1000 h. All the SM-OPV devices exhibited excellent stability under the thermal stress (Figure S1 of Supporting Information).

The photostability of the SM-OPV devices was investigated by light soaking them under one sunlight for 1500 h (denoted as L in Figure 2b). The one sunlight was illuminated through a 450 nm-long pass filter to exclude the effect of the dimerization of PC_70_BMs by UV light. A substantial degradation of the solar cell performance was observed when the devices were subjected to light soaking (Figure 2b and Table 1). Among the three types of devices, Binary (L) was the least stable and Ternary-F (L) was the most stable. The PCE of Binary (L) decreased to 0.87% after light soaking for 1500 h, which corresponds to 17% of the initial PCE.

**Figure 3:**
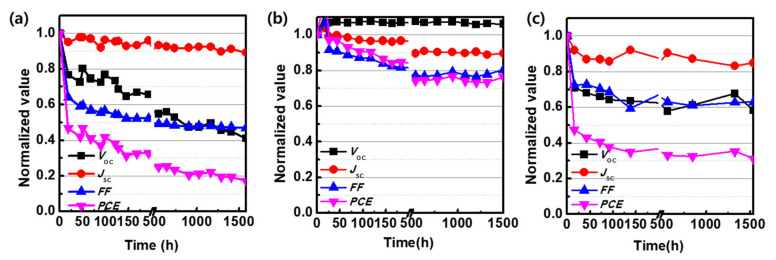
Changes in the solar cell parameters of (**a**) Binary, (**b**) Ternary-F, and (**c**) Ternary-CN as a function of the light-soaking time.

Plots of solar cell parameters as a function of the light-soaking time (Figure 3a) indicate that the instability of Binary (L) was mainly because of the rapid reduction in *V*oc and *FF* within 50 h of light soaking (initial burn-in loss). Although the initial burn-in loss of Binary (L) slightly improved following the addition of amorphous PPDT2CNBT to LGC-D073:PC_71_BM (Ternary-CN (L)), the burn-in loss was still severe (Figure 3c). Ternary-CN (L) exhibited a PCE of 1.61% after light soaking, which was 31% of the initial PCE. As in the case of Binary (L), the rapid reduction in *V*oc and *FF* was the main cause of the initial burn-in loss of Ternary-CN (L). The initial burn-in loss was considerably improved by the addition of semi-crystalline PPDT2FBT to LGC-D073:PC_71_BM (Ternary-F (L)). Ternary-F (L) retained 76% of its initial PCE (Figure 3b) and showed a PCE of 3.84% after light soaking for 1500 h. Compared to Binary (L), the rate of decrease in *J*sc and *FF* of Ternary-F (L) was significantly reduced and, more importantly, the *V*oc of Ternary-F (L) remained constant during the light soaking for 1500 h.

**Figure 4:**
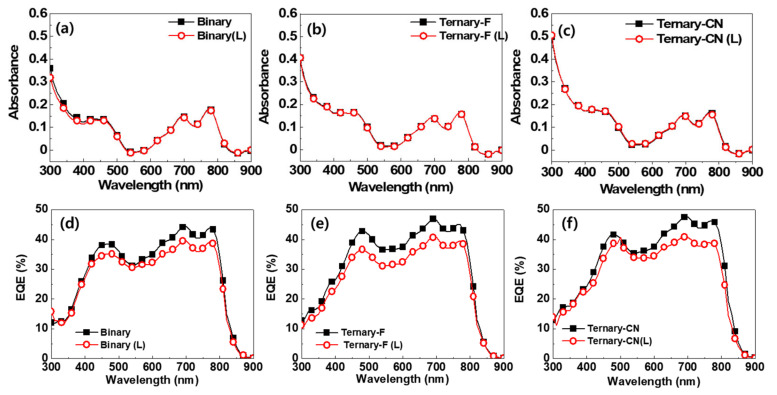
Comparison of the absorption spectra (**a**–**c**) and EQE spectra (**d**–**f**) of Binary, Ternary-F, and Ternary-CN before and after the light-soaking test.

It has been reported that the photodegradation of the active layer can occur as a result of photochemical processes triggered by UV radiation, leading to absorbance and *V*_OC_ loss [32]. There was no change in the absorption spectra of all the photoactive layers after light soaking (Figure 4a–c). Because the devices were sealed with glass cap and epoxy resin in the nitrogen filled glove box, it is considered that the photooxidation was not occurred during the test. Therefore, the reduction in *V*_OC_ is not related to the photo-oxidation of the photoactive layers. A slight decrease in the EQE was observed for all the light-soaked SM devices (Figure 4d–f), and this decrease shows a good agreement with the decrease in *J*_SC_ after light soaking. Considering the steps in determining the EQE of a solar cell, exciton diffusion, charge dissociation and charge collection steps must be related to the reduction in EQE of light-soaked devices rather than the absorption step [33].

**Figure 5:**
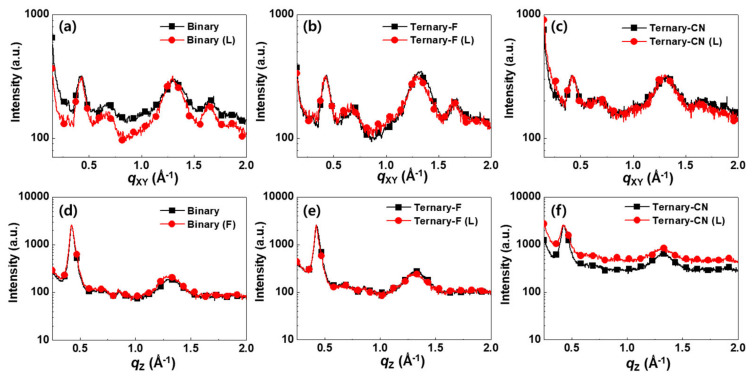
In-plane (**a**–**c**) and out-of-plane (**d**–**f**) Grazing incidence wide angle X-ray scattering (GIWAX) line-cut profiles of Binary, Ternary-F, and Ternary-CN films before and after the light-soaking test.

Grazing incidence wide-angle X-ray scattering (GIWAXS) experiments were conducted to investigate the BHJ morphology of the photoactive layers (Figure 5). Peaks at around 0.42 and 1.6 Å^−1^ are attributed to the lamella and π–π staking of the LGC-D073 molecules, respectively. In addition, a peak from PC_71_BM stacking was observed at around 1.3 Å^−1^. Detailed peak information and two-dimensional images are presented in Table S1, Table S2, and Figure S2 of Supporting Information. The Binary film showed a strong lamella peak in the *q_xy_* direction, and a π–π staking peak was clearly observed in the *q_z_* direction, which indicated that LGC-D073 had a face-on orientation in the Binary film. The addition of PPDT2FBT (Ternary-F) or PPDT2CNBT (Ternary-CN) did not alter the packing direction of the LGC-073 molecules. However, the scattering intensity of the π–π stacking of Ternary-CN was smaller than those of Binary and Ternary-F (Figure 5a–c). This implies that the addition of amorphous PPDT2CNBT to the photoactive layer might disturb the ordered LGC-D073 domains and the optimized BHJ structure, which may be related to the low *FF* of Ternary-CN. However, the peaks of all the films did not show any noticeable change after light soaking for 300 h. This implies that no significant morphological changes were induced during light soaking in the photoactive layer within the detection limit of the GIWAXS experiments.

**Figure 6:**
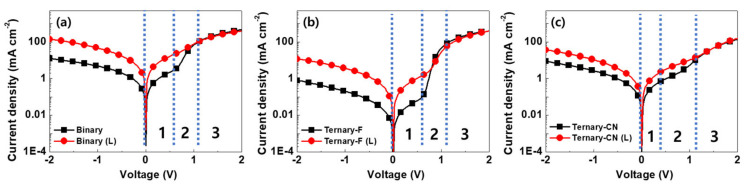
*J-V* characteristics of (**a**) Binary, (**b**) Ternary-F, and (**c**) Ternary-CN before (solid square) and after (solid circle) the light-soaking test.

The *J–V* characteristics under dark conditions are shown in Figure 6. The rectification ratio of Ternary-F was 478 at ±2 V, and this value was significantly larger than the values of Ternary-CN (16) and Binary (37). Although the rectification values decreased after light soaking for all the devices, Ternary-F maintained the highest rectification ratio among the devices. The higher rectification ratio and lower leakage current of Ternary-F and Ternary-F (L) indicate that Ternary-F is closer to the ideal diode compared to the Ternary-CN and Binary.

The shunt resistance was determined at *V* = 0—namely, 1/*R*_sh_ = −(*dJ*/*dV*)*_J_*_= *J*sc_ (region 1 in Figure 6) [34]—and the series resistance was determined at *J* = 0—namely, 1/*R*_s_ = −(*dJ*/*dV*)*_V_*_= *V*oc_ (region 3 in Figure 6). The calculated *R_s_* values of the devices were similar in the range of 11–24 Ω. However, the *R*_sh_ values differed considerably (Table 1). The *R_sh_* value of Ternary-F was calculated to be 94,426 Ω, which is 66 and 28 times larger than those of Binary (1428 Ω) and Ternary-CN (3372 Ω), respectively. After light soaking, the *R*_sh_ values of Binary (L), Ternary-CN (L), and Ternary-F (L) decreased considerably to 187, 1089, and 5968 Ω, respectively. However, it is noticeable that Ternary-F (L) maintained high *R*_sh_ values even after light soaking, implying that the leakage current can be prevented more efficiently in this device compared to the cases of Ternary-F(CN) and Binary.

Bimolecular recombination and trap-assisted recombination are the main nongeminate recombination mechanisms [35,36,37]. In OPV devices, nongeminate recombination can be investigated by analyzing the *J*_SC_ or *V*_OC_ as a function of the light intensity. *J*_SC_ is known to follow a power-law dependence on the light intensity (*I*), which can be described as *J*_SC_ ∝ (*I*)*^S^*, where *S* is an exponential factor. Bimolecular recombination weakens as the value of *S* approaches 1 [38]. The plots of log (*J*_SC_) versus log (*I*) for all the devices exhibited a linear relationship, and the *S* values of all devices before and after light soaking were in the range of 0.97 and 1.04 (Figure S3 and Table S3). This indicates that bimolecular recombination is not a major recombination mechanism in these SM-OPV devices.

The degree of trap-assisted recombination (Shockley–Read–Hall recombination) in solar cell devices can be evaluated from the light intensity dependent *V*_OC_ measurements. This dependence can be expressed as:

(1) $$V_{OC}=\;\left(\frac{k_{b}T}{q}\right)\ln\left(I\right)+constant,$$

where *k_b_* is the Boltzmann constant, *T* is the temperature, *q* is the elementary charge, and *I* is the light intensity. The slope in the plot of *V*_OC_ versus ln(*I*) is known to approach *k_b_T*/*q* when bimolecular recombination (Langevin recombination) is dominant. A stronger dependence on light intensity is expected in the presence of trap-assisted recombination, showing a slope greater than *k_b_T*/*q* [37,39,40].

**Figure 7:**
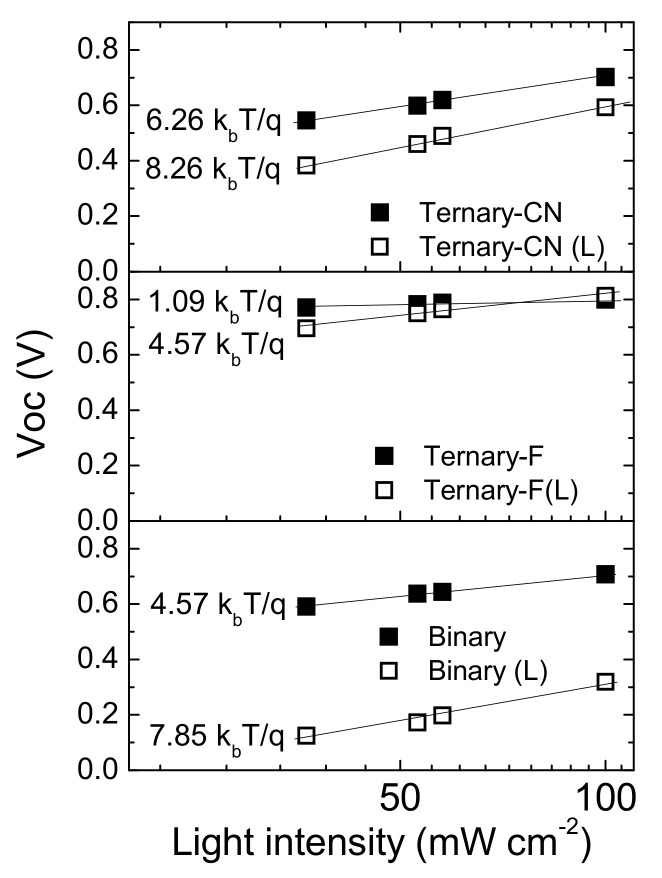
Light intensity vs. *V*_OC_ plots for Binary, Ternary-F, and Ternary-CN before and after the light-soaking test.

As shown in Figure 7, the slope values for Binary and Ternary-CN deviated significantly from *k_b_T*/*q*, known as ideality factor (*n*). For the ideal devices with Langevin recombination, the value approaches unity. The large *n* value of the Binary and the Ternary-CN suggests that recombination in open-circuit conditions is dominated by charge-carrier trapping [39,40,41]. Mandoc et al. showed that the introduction of 7,7,8,8-tetracyanoquinodimethane electron traps in MDMO-PPV: PC_71_BM blends strongly modifies *V_OC_* and its dependence on the light intensity (e.g., the slope of *S* changes from 1.05 *kT*/*q* to 3.04 *kT*/*q*) [41]. On the other hand, the slope of Ternary-F was close to *k_b_T*/*q*, indicating that the trap density of the device was effectively reduced by the addition of a small amount of PPDT2FBT. After light soaking, the slope values of all devices increased considerably. However, the value for Ternary-F (L) was significantly lower than those for Ternary-CN (L) and Binary (L).

Transient photovoltage (TPV) measurements also provide information on the trap-assisted recombination in devices [42,43,44,45]. The TPV technique was used to measure the recombination lifetime (*τ*_rec_) as a function of the current density. The parameter *τ*_rec_ was extracted by plotting the decay of *V*_OC_ as a function of time and fitting the curve with a mono-exponential decay function. We measured the TPV at the *V*_OC_ condition for different light intensities. When the device output reached a steady state, pulsed light was applied to generate a small perturbation. Because no charge is collected in the open-circuit condition, the excess charge carriers generated by the pulsed light recombine with the lifetime τ_rec_, resulting in the decay of *V*_OC_. It has been reported that nongeminate recombination is closely related to the decrease in V*_OC_*.

**Figure 8:**
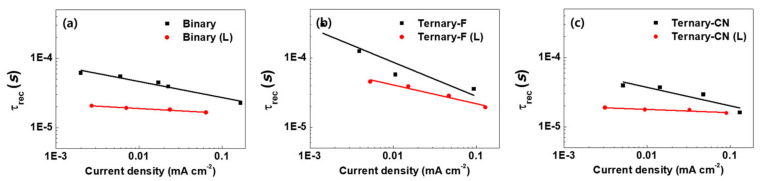
Plots of recombination lifetime vs. current density for (**a**) Binary, (**b**) Ternary-F, and (**c**) Ternary-CN before and after the light soaking.

As shown in Figure 8, Ternary-F exhibited a longer τ_rec_ compared to those of Ternary-CN and Binary, implying that Ternary-F had the least trap-assisted recombination. After light soaking, all the τ_rec_ values for all devices decreased substantially. However, Ternary-F (L) exhibited longer τ_rec_ values compared to those of Ternary-CN (L) and Binary (L). As the formation of localized trap states promotes trap-assisted recombination and reduces *V*_OC_, it appears that the addition of PPDT2FBT reduced trap-assisted recombination and led to a stable *V*_OC_ in Ternary-F during the light-soaking test [46,47].

As explained above, hopping is the rate-determining step for charge transport in SM-OPVs, and an increased hopping-mediated charge transport increases the chances of charge trapping. Therefore, charge transport between LGC-D073 domains would be critically dependent on the degree of molecular orientation and morphological change. This implies that a subtle change in the molecular morphology by light soaking could significantly alter the charge-carrier mobility in SM-OPVs. Such subtle changes may not be detected by GIWAXS experiments. The addition of a CP to the LGC-D073:PC_71_BM blend could provide an efficient percolation pathway for charge transport between the LGC-D073 domains and render LGC-D073-based SM devices less sensitive to subtle morphological changes.

**Figure 9:**
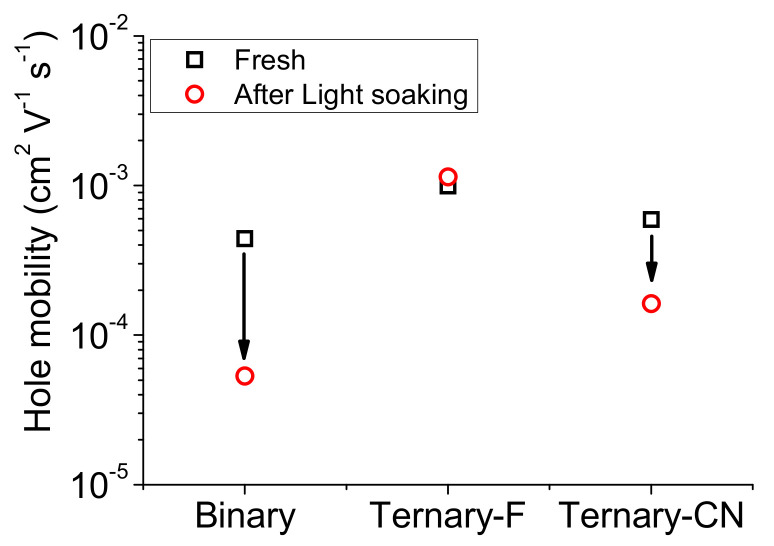
Changes in the hole mobility for Binary, Ternary-F, and Ternary-CN after light soaking.

The hole mobilities of the SM-OPV devices before and after light soaking were determined using the space charge limited current model, and the results obtained are shown in Figure 9. The hole mobility value of the as-prepared Ternary-F was 2.3 times higher than that of Binary. As expected, the addition of crystalline PPDT2FBT to the LGC-073:PC_71_BM blend effectively increased the hole mobility of the Ternary-F film. However, the addition of PPDT2CNBT to the LGC-073:PC_71_BM blend was not as effective as that of PPDT2FBT. The hole mobility of Ternary-CN was 1.4 times higher compared to that of Binary. Although the addition of PPDT2CNBT led to the formation of percolation pathways for hole transport, it seems that it disturbed the formation of ordered LGC-D073 domains. After light soaking for 50 h, the hole mobility of Ternary-F remained almost unchanged. However, the hole mobility of Ternary-CN and Binary decreased considerably after the light-soaking test. After the test, the hole mobilities of Ternary-CN and Binary were reduced by 8.3 and 3.7 times, respectively. These results indicate that the percolation pathway formed upon the addition of PPDT2FBT maintained the hole mobility of Ternary-F constant even after light soaking.

## 4. Conclusions

The photostability of SM-OPV devices was greatly improved by the addition of semi-crystalline CP because of the formation of percolation pathways for charge transport without disturbing the optimized BHJ structure. However, the addition of amorphous CP did not improve the photostability of the devices because it inhibited the formation of ordered SM domains. This result suggests that the crystallinity of the CP additive is important for improving the stability of SM-OPV devices.

## Figures and Tables

**Table 1:** Solar cell parameters of the fabricated SM-OPV devices before and after 1 sunlight soaking for 1500 h.

Device	*V*_OC_ (V)	*J*_SC_ (mA·cm^−2^ )	*FF*	Best Eff. (%)	Avg. Eff. (%)	*R*_s_ (Ω)	*R*_sh_ (Ω)
Binary	0.75	11.64	0.59	5.17	4.85	11.07	1428
Binary (L) *	0.31	10.46	0.27	0.87	0.50	13.91	187
Ternary-F	0.80	12.17	0.63	6.09	5.94	19.82	94,426
Ternary-F (L)	0.79	10.23	0.48	3.84	3.77	18.56	5968
Ternary-CN	0.84	11.65	0.54	5.29	4.95	23.67	3372
Ternary-CN (L)	0.49	9.89	0.34	1.61	1.33	19.17	1089

* (L) denotes device after light soaking for 1500 h.

## Supplementary Materials

The following are available online at https://www.mdpi.com/2073-4360/12/11/2598/s1, Figure S1: Thermal stability of SM-OPV devices. All the devices were subjected to thermal stress at 80 °C for 1000 h. Figure S2: 2D images of GIXRD as prepared (a) Binary, (b) Ternary-F, and (c) Ternary-CN films and light soaked (d) Binary, (e) Ternary-F, and (f) Ternary-CN films. Figure S3: Plots of log (*J*_SC_) versus log (*I*) for all the devices. Table S1: In-plain π-π stack peak information of Binary, Ternary-F, and Ternary-CN films before and after light soaking. Table S2: Out-of-plain lamellar stack peak information of Binary, Ternary-F, and Ternary-CN films before and after light soaking. Table S3: The *S* values of all devices before and after light soaking.
