# Supplementary material for: Improving the Photostability of Small-Molecule-Based Organic Photovoltaics by Providing a Charge Percolation Pathway of Crystalline Conjugated Polymer
Source: Polymers (Basel). 2020 Nov 5;12(11):2598. doi: 10.3390/polym12112598 (PMC7694356; doi:10.3390/polym12112598)
Supplement: Supplementary file 1 [file polymers-12-02598-s001.pdf]

Supplementary Materials

Improving the Photostability of Small-Molecule-Based Organic Photovoltaics by Providing a Charge Percolation Pathway of Crystalline Conjugated Polymer

Jihee Kim [1], Chang Woo Koh [2], Mohammad Afsar Uddin [2], Ka Yeon Ryu [1], Song-Rim Jang [3], Han Young Woo [2,*], Bogyu Lim [3,4,*] and Kyungkon Kim [1,*]

[1] Department of Chemistry and Nano Science, Ewha Womans University, Seoul 03760, Korea; jhkim3664@ewhain.net (J.K.); ryuky@ewhain.net (K.Y.R.)
[2] Department of Chemistry, Korea University, Seoul 136713, Korea; woocchang@korea.ac.kr (C.W.K.); soaibchebd@yahoo.co.uk (M.A.U.)
[3] Future Technology Research Center, LG Sciencepark, LG Chem, 30, Magokjungang 10-ro, Gangseo-gu, Seoul 07796, Korea; songrimjang@lgchem.com
[4] Green Fine Chemical Research Center, Advanced Convergent Chemistry Division, Korea Research Institute of Chemical Technology (KRICT), 45 Jongga-ro, Jung-gu, Ulsan 44412, Korea
[*] Correspondence: hywoo@korea.ac.kr (H.Y.W.); bglim@krict.re.kr (B.L.); kimkk@ewha.ac.kr (K.K.)

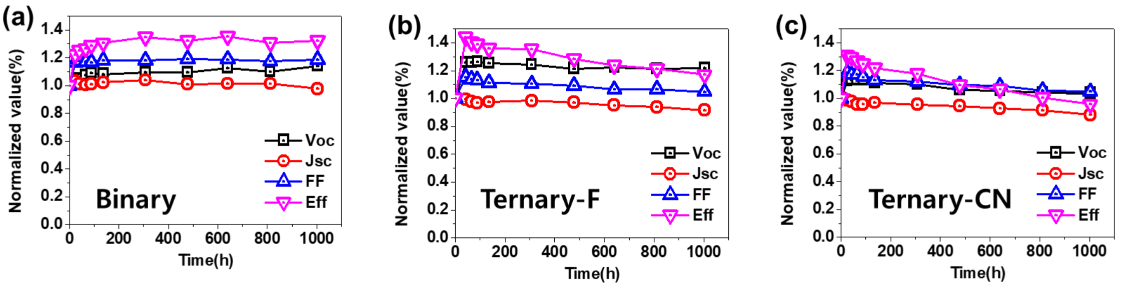

Figure S1. Thermal stability of SM-OPV devices. All the devices were subjected to thermal stress at 80°C for 1000h.

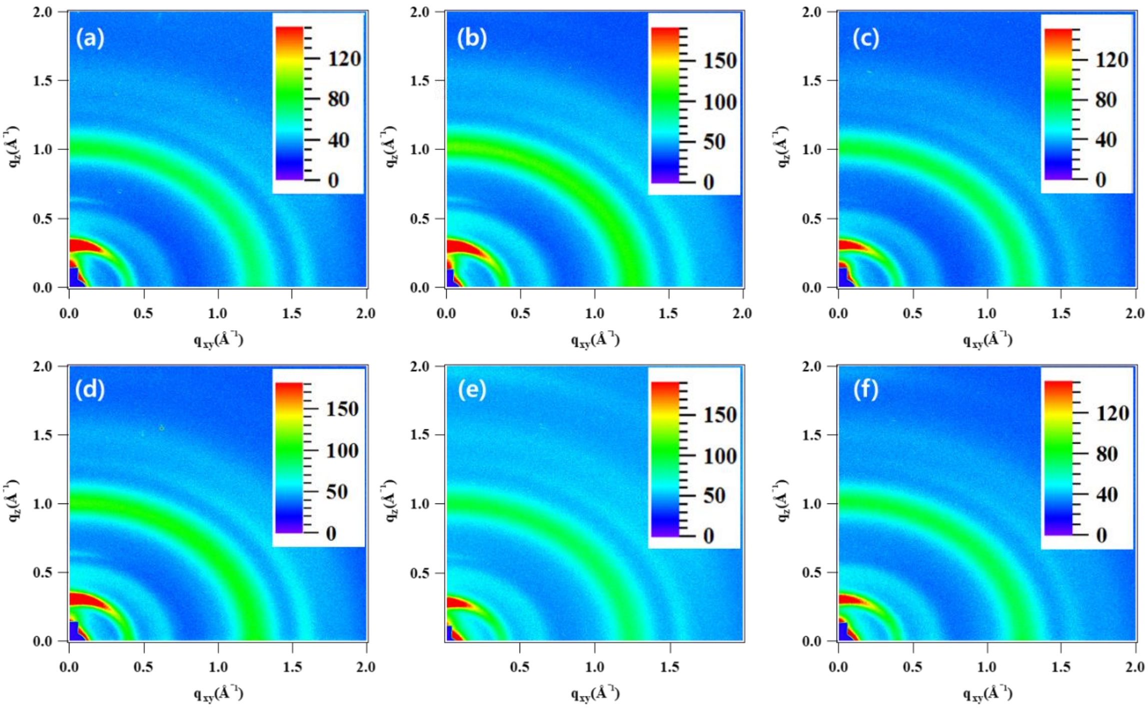

Figure S2. 2D images of GIXRD as prepared (a) Binary, (b) Ternary-F and (c) Ternary-CN films and light soaked (d) Binary, (e) Ternary-F and (f) Ternary-CN films.

Table S1. In-plain π-π stack peak information of Binary, Ternary-F and Ternary-CN films before and after light soaking.

Direction	Material	(010) π-π stack		
		Scattering vector(q) [$Å^{-1}$]	d-spacing [Å]	FWHM
**In-plane**	Binary	1.689	3.720	0.116
	Binary(L)	1.664	3.775	0.107
	Ternary-F	1.677	3.747	0.088
	Ternary-F(L)	1.630	3.855	0.107
	Ternary-CN	1.622	3.874	0.155
	Ternary-CN(L)	1.634	3.845	0.159

Table S2. Out-of-plain lamellar stack peak information of Binary, Ternary-F and Ternary-CN films before and after light soaking.

Direction	Material	(200) Lamellar stack		
		Scattering vector(q) [$Å^{-1}$]	d-spacing [Å]	FWHM
Out - of plane	Binary(D)	0.425	14.783	0.0349
	Binary(L)	0.423	14.861	0.0391
	Ternary-F(D)	0.427	14.706	0.0320
	Ternary-F(L)	0.425	14.783	0.0307
	Ternary-CN(D)	0.427	14.706	0.0568
	Ternary-CN(L)	0.427	14.706	0.0693

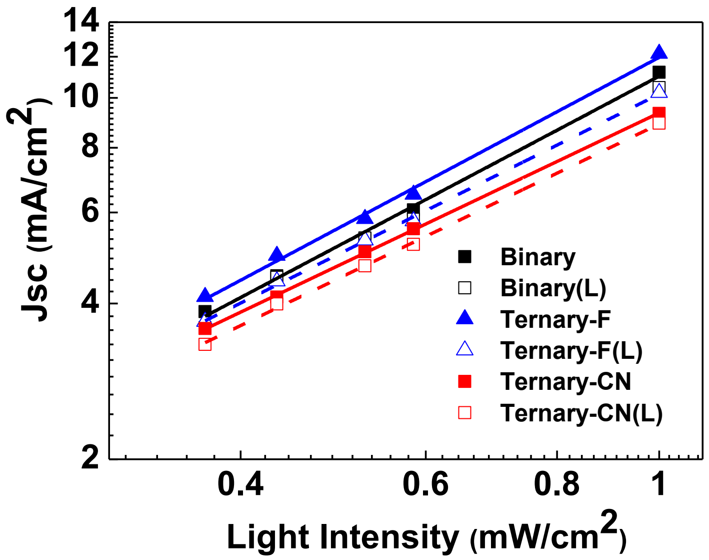

Figure S3. Plots of log (J_{SC}) versus log (I) for all the devices.

Table S3. The S values of all devices before and after light soaking.

	S value	Standard deviation
Binary	1.04	0.025
Binary(L)	1.02	0.037
Ternary-F	0.96	0.009
Ternary-F(L)	0.98	0.008
Ternary-CN	0.97	0.009
Ternary-CN(L)	0.98	0.018
